# Editorial: Advances in haptic feedback for neurorobotics applications

**DOI:** 10.3389/fnins.2023.1189749

**Published:** 2023-04-11

**Authors:** Min Li, Emanuele Lindo Secco, Yang Zheng, Chenyun Dai, Pengwen Xiong, Guanghua Xu

**Affiliations:** ^1^Institute of Engineering & Medicine Interdisciplinary Studies, School of Mechanical Engineering, Xi'an Jiaotong University, Xi'an, China; ^2^School of Mathematics Computer Science & Engineering, Liverpool Hope University, Liverpool, United Kingdom; ^3^Center for Biomedical Engineering, School of Information Science and Technology, Fudan University, Shanghai, China; ^4^School of Advanced Manufacturing, Nanchang University, Nanchang, Jiangxi, China

**Keywords:** neural-machine interfaces, neurorobotics, motion intention decoding, haptic sensing, haptic feedback

Neuro-robots and neural-machine interfaces have shown to offer a set of solutions and benefits on a variety of disciplines, including assistive and rehabilitative devices for individuals with motor dysfunction, telerobotics, and good Human robot interaction with prosthetics.

Typically, the interaction between these systems and the end-user is performed by detecting the human intention through the acquisition of physiological parameters or action, such as, for example, the ElectroEncephaloGram (EEG), the ElectroCardioGram (ECG), the ElectroMyoGram (EMG), ElectroOculoGram (EOG), or the movement of a limb.

In this context we have launched this novel Research Topic on “*Advances in haptic feedback for neurorobotics applications”* where, in particular, we want to focus on the human-machine interaction from a haptics viewpoint.

The reason why we have introduced Haptic feedback is twofold: first, we believe that is crucial to have robotics-designs which are *human-centered*, i.e., they are defined, developed, and improved around the subject and the end-user. Secondly, but not less important, we also strongly believe that *Haptic Feedback* is at present still under-estimated and not sufficiently investigated as a real-time and intuitive mean between the robotic device and the human being.

Accordingly, our call has received a nice set of answers and the Research Topic has collected the interest of more than 60 authors around the word, presenting an intriguing set of human-centered original researches.

## Novel motion intention decoding methods and algorithms

Introducing novel motion intention decoding methods and exploring novel algorithms with good robustness and the reliability is a very important aspect of this Research Topic. Li et al. presented “*A novel EEG decoding method for a facial-expression-based BCI system using the combined convolutional neural network and genetic algorithm”* where they showed that their Facial-Expression-based BCI system provides superior performance vs. traditional methods.

Gesture recognition can also be used for motion intention decoding. Ruan et al. looked at how PhotoPlethysmoGraphy signals may improve gesture recognition vs. human-computer interaction performed by means of wearable devices and they presented wrist- and finger-related gesture recognition based on “*Principal component analysis of photoplethysmography signals for improved gesture recognition”*.

Zhang et al. looked at motion intention decoding in human-robot interaction from a further perspective and they presented a Personalized Speed Adaptation method where EEG and EOG capture operator's mental state, and then regulate robot's speed according to this mental state. To the best of our knowledge, this paper is the first “*Feasibility study of personalized speed adaptation method based on mental state for teleoperated robots”*.

## Haptic feedback modalities

Haptic feedback modalities are another important direction of this Research Topic. Electro-tactile feedback is a common haptic feedback modality for prosthesis. The dexterous movements of the upper limbs are inseparable from proprioceptive feedback. Han et al. showed how prosthetic sensory feedback could benefit of the “*Substitutive proprioception feedback of a prosthetic wrist by electro-tactile stimulation”* in experiments with five able-bodied subjects and two amputee subjects. Vibrotactile feedback is another common haptic feedback modality for prosthesis.

How to choose the location of tactile feedback in amputee users is an important question to be answered. Morand et al. proposed a “*FeetBack–Redirecting touch sensation from a prosthetic hand to the human foot”* where a vibrotactile insole was set up in order to vibrate according to the sensed force of prosthetic fingers while subjects manipulate fragile and heavy objects, providing a novel approach vs. tactile sensation in myoelectric prosthetics.

In a similar context, Pardo et al. investigated vibrotactile sensation of the arm-shoulder region in “*Vibrotactile mapping of the upper extremity: absolute perceived intensity is location-dependent; perception of relative changes is not,”* providing an overview of the sensory bandwidth that can be achieved with vibrotactile stimulation of the human arm. The result may help in the design of vibrotactile feedback interfaces (displays) for the hand/arm/shoulder-region.

Evaluating other possible haptic feedback modalities rather than commonly-used ones is another research direction. Mayer et al. looked at the “*Temporal and spatial characteristics of bone conduction as non-invasive haptic sensory feedback for upper-limb prosthesis”*, highlighting this approach's potential as a non-invasive feedback modality for upper-limb prostheses.

## Control strategies

In order to improve the utility and user experience of neuro-robots, robust closed-loop control with respect to disturbance is needed. On the “*EMG feedback outperforms force feedback in the presence of prosthesis control disturbance”*, Tchimino et al. showed that EMG feedback may provide better performance vs. force feedback in human-prosthesis interaction.

Another interesting contribution for this direction was coming from Bruni et al., where the authors validate an “*Object stiffness recognition and vibratory feedback without ad-hoc sensing on the Hannes prosthesis by means of a machine learning approach.”* The experimental results proved that the proposed strategy allowed able-bodied subjects and amputees to recognize the objects' stiffness accurately and quickly.

To sum up, this Research Topic aims to highlight the most advanced achievements in motion intention decoding, haptic sensing, and haptic feedback for Neural-Machine Interface (NMI)-based neurorobotics research, which can be applied for teleoperation, human robot interaction with prosthetics, assistive and rehabilitative robots, and other relevant circumstances. The novel neural decoding methods, novel haptic feedback modalities, and new control strategies reported in this Research Topic should inspire and guide the future direction of this field.

## Author contributions

ES and ML: writing. YZ, GX, CD, and PX: modification. All authors contributed to the article and approved the submitted version.

